# Free and Immobilised β-Glucosidases in Oenology: Biotechnological Characterisation and Its Effect on Enhancement of Wine Aroma

**DOI:** 10.3389/fmicb.2021.723815

**Published:** 2021-08-09

**Authors:** Pilar Fernández-Pacheco, Beatriz García-Béjar, Ana Briones Pérez, María Arévalo-Villena

**Affiliations:** ^1^Food Technology Department, Faculty of Environmental Science and Biochemistry, Castilla-La Mancha University, Toledo, Spain; ^2^Analytical Chemistry and Food Technology Department, Faculty of Chemical Sciences and Technologies, Castilla-La Mancha University, Ciudad Real, Spain

**Keywords:** β-glucosidases, immobilised enzyme, monoterpenes, aroma precursors hydrolysis, wine aroma

## Abstract

In grapes, monoterpenes and norisoprenoids are in the form of non-volatile compounds, flavourless glycosides which could enhance the aroma of wines after its hydrolysis using β- glucosidases enzymes. It is known that the use of immobilised enzymes offers advantages such as reusability and easy recuperation. In this study, a commercial β-glucosidase was immobilised by absorption in sodium alginate. Biotechnological characteristics and terpen hydrolysis (hydrolysis aroma precursors) in muscat wines were studied after treatment with both free and immobilised commercial β- glucosidase with two different concentrations. It was revealed that both forms shared an optimal pH (4.5) and a maximum temperature (64°C), even an increment on the activity between 40and 60°C. A similar Km value has been determined while Vmax from the immobilised enzyme was higher than the free (3.35 and 2.52 μmol min^–1^ mg^–1^, respectively). Additionally, the immobilised enzyme showed a better hydrolytic activity during 24 h, and its reusability has been proven. Regarding enzymatic hydrolysis in grape must, the best results were observed for the highest concentration of free β-glucosidase although glucose release was also determined for the immobilised enzyme along the days. In contrast, maximum activity was reached by the immobilised β-glucosidase in less time but in no case equalled the free ones. Finally, volatile compound liberation in wines treated with free or immobilised enzymes was analysed using HRGC-MS. Liberation for both enzymes and the greatest concentrations of some volatiles were detected when a double dose of the free β-glucosidase was used. Nevertheless, the wines treated with the immobilised β-glucosidase showed a high concentration of some volatile compounds such as nerol or geraniol.

## Introduction

Monoterpenes and norisoprenoids from grapes are important aroma compounds, which improve flavour in wines, especially white wine. Reports indicate that not all glycosides are present in all grape varieties and that concentrations vary across them (Bayonove et al., 1993). In this way, Muscat, Gewürztraminer, Riesling, or Sauvignon Blanc are varieties with higher concentrations in these compounds.

In addition, grapes also include compounds called “aroma precursors,” the most active of which are glycosides—mostly linalool, nerol, and geraniol (Alem et al., 2018). These compounds are usually present in two fractions: (i) a free fraction, which contributes directly to must aroma, and (ii) a bound fraction, which forms non aromatic glycosides (these glycosides may later release their aromatic load). The bound fraction is quantitatively more significant than the free fraction since several potential aroma compounds exist as non-volatile compounds, flavourless glycosides in grapes (Gunata et al., 1985; Ghaste et al., 2015). The release of glycosidically bound aroma compounds by acid or enzyme treatment can enhance the total aroma of processed fruit products and aroma wines (Arévalo-Villena et al., 2006a).

Monoterpenes are linked to β-D-glucose units or disaccharides containing glucose and a second sugar, such as a-arabinose, α-rhamnose, and β-apiose. The sequential mechanism of enzymatic hydrolysis is now well established: in the first step, glycosidic linkage is cleaved by either a α-arabinofuranosidase (Ara), a α-rhamnosidase (Rha), or a β-apiofuranosidase (Apio), and then a β-glucosidase (BG) liberates the monoterpenols (Gunata et al., 1988; González-Pombo et al., 2014).

Aroma enhancement of wines using β-glucosidase enzymes has been researched by some authors. Glycosidases from Aspergillus niger and some Saccharomyces and non-Saccharomyces species such as Debaryomyces hansenii, D. pseudopolymorphus, Hanseniaspora uvarum, among others, have been found to possess interesting properties for practical use in winemaking (Arévalo-Villena et al., 2007; Mateo and Maicas, 2016).

While the effect of this enzyme has been reported widely, the use of immobilised enzymes in winemaking is still at the experimental stage and are still used in their free form.

Nevertheless, in recent years some studies on immobilisation β-glucosidase have been carried out, and it is noticeable that it can be used in enology as a valid alternative for winemaking and could provide substantial advantages to the sector.

Arévalo-Villena et al. (2006a) found that the use of supported enzymes was feasible to hydrolyse the aromatic precursors of Muscat. In addition, glucosidase immobilisation allows for a precise management of the conversion degree, achieving a rapid and controlled liberation of terpenes while preserving a fraction of bound aromas as a reserve to be released along the time (González-Pombo et al., 2011).

Some studies show their potential: Romo-Sánchez et al. (2014) immobilised a commercial β-glucosidase in the chitosan-chitin matrix and showed exciting results regarding the release of monoterpenes bound in Muscat wines and its reusability (enzyme reuse through multiple cycles of batch fermentation processes resulted in significant cost savings). Alternatively, β-glucosidase immobilisation (Eupergit C) has shown high ability to liberate norisoprenoids and phenols from their precursors in Cabernet Sauvignon wine (de Ovalle et al., 2018).

Immobilised enzymes present the advantage of being easy to recover after use. A further benefit of this kind of enzymes is that they are often more stable and resistant to environmental conditions and easier to handle (Liu et al., 2018). In addition, β-glucosidase immobilisation enables an accurate management of the conversion degree, achieving a rapid and controlled liberation of terpenes. This favours a quick sale while preserving a fraction of bound aromas to be released over time (González-Pombo et al., 2011).

Considering the interest in stable/immobilised enzymes for industrial use and the importance of enhancing aroma wine and juices, the aim of this study was to immobilise commercial β-glucosidase on sodium alginate by microencapsulation. Meanwhile, the immobilised enzyme was characterised (optimal conditions of pH, T^a^, and maximum activity), and kinetic parameters and reusability were studied. Finally, it quantified the hydrolysis of aroma precursors from isolate Muscat glycosides and the enhancement of aroma directly in wines and compared them with free commercial enzymes.

## Materials and Methods

### β-Glucosidase Immobilisation

The chosen enzyme was a commercially soluble β-glucosidase (Lallemand, Spain) because of its importance in winemaking. Sodium alginate from Laminaria hyperborea was the support used for the immobilisation process (BDH, Poole, United Kingdom).

β-glucosidase was mixed with 3 mL sodium alginate (5.0%) in a final volume of 10 mL (0.1 g enzyme/mL; amount recommended by the manufacturer). Then, the mixed suspension was dropped into a CaCl_2_ solution (50 mM). After 10–15 min, the calcium-alginate beads were washed twice with distillate water to remove the excess CaCl_2_.

### Ascertain of β-Glucosidase Activity

Hydrolysis of cellobiose and quantification of released glucose were conducted to study the β-glucosidase activity of free and immobilised enzymes. The glucose was analysed using an enzymatic test (Glucose Go) as described in Arévalo-Villena et al. (2006b).

A solution of the commercial β-glucosidase (100 μL) was mixed with 400 μL of 1% (w/v) D-(+)-cellobiose in a 50 mM citrate-phosphate buffer (pH 5.5) incubated at 37°C for 30 min. The assays were done in triplicate, and results were given as units of enzyme (U), where one unit is defined as the amount of enzyme required to release one mol of glucose per minute under the abovementioned assay conditions.

### Biotechnological Characterisation of Free and Immobilised β-Glucosidase

The influence of diverse parameters such as pH, temperature, and hydrolytic capability on enzymatic activity in both free and immobilised β-glucosidase was studied. Meanwhile, the kinetic constants and reusability of the immobilised enzyme were also determined.

#### pH Stability

Enzymes were incubated in the presence of cellobiose at 37°C for 30 min. Moreover, different pH values (2.5–7.0) were set using different buffers (100 mM citric acid/Na2HPO4 for pH 2.0–2.5, 100 mM sodium acetate/acetic acid for pH 3.0–6.0, and 100 mM Na2HPO4/NaH2PO4 for pH 6.5–8.0).

#### Optimum Temperature of Hydrolysis

The enzymes were maintained in a temperature range between 13 and 80°C at their optimum pH, and the release of glucose was studied under the described conditions above.

#### Hydrolytic Capability Along the Time

The release of glucose was studied for 24 h. For this purpose, enzymatic activity was analysed for each hour during the first 8 h and finally to 24 h.

#### Kinetic Constants

Vmax (μmol min^–1^ mg^–1^) and kinetic constant Km (mM) were calculated from the Michaelis–Menten plots of specific activities at 0.025 to 1% concentrations of substrate. The rates were also measured, ranging from 0.2 to 5 times the value of Km, and the values of Vmax and Km were determined by non-linear regression (Arévalo-Villena et al., 2006c).

#### Reusability of Immobilised Enzyme

Immobilised β-glucosidase activity was evaluated in consecutive cycles and reused in the same conditions as the previous reaction. Immobilised enzyme was washed with acetate buffer (pH 3.5; 50 mM) between consecutive reactions to remove any residual substrate. Activity was assessed by quantifying the hydrolysis of cellobiose.

### Enzymatic Hydrolysis of Aroma Precursors in Grape Must

Immobilised and free enzymes were used at two different concentrations: the reference, 0.026, and the half, 0.013 mg/L.

The method proposed by Arévalo-Villena et al. (2006b) and Romo-Sánchez et al. (2014) was used for extracting the glycosylated compounds from Muscat must. Briefly, compounds were obtained by retention and subsequent elution in C18 columns. Then, an aliquot of the extract (400 μL) was mixed with 100 μL of the free or immobilised enzyme and incubated for 12 days at 37°C. The liberated glucose was analysed, and the number of free precursors was compared with the total amount of precursors obtained after total acid hydrolysis since the process is carried out in an equimolecular manner.

### Application of Immobilised and Free β-Glucosidase in White Wine

#### Microvinification

Muscat must variety from Castilla – La Mancha (Spain), supplied with 50 ppm of SO2, was inoculated with 106 cells/mL of a commercial Saccharomyces bayanus strain (CECT 1996) and microfermented at 18°C by duplicated. Glucose and fructose contents were determined by HPLC. After the fermentation process, wines were decanted and fractionated for the next assay.

#### Aroma Precursors Hydrolysed by Free and Immobilised β-Glucosidase

Immobilised and free enzymes were added to wine fractions (30 mL) in two concentrations: 0.026 mg/L (recommended dose in a previous study) and 0.013 mg/L to determine whether the half dose provides similar results. The treatments were carried out in triplicate at 20°C for 16 days, and bentonite (20 g/hL) was added to remove the enzymes. Afterward, the wine samples were centrifuged and kept at 4°C until analysis.

### Quantification of Volatile Compounds by HRGC-MS Analysis

#### Isolation of Volatiles and Terpenic Compounds; Solid-Phase Extraction Using ENV + Cartridge

Terpenic compounds were obtained using the solid-phase extraction (SPE) technique after their adsorption and a separate elution using an Isolute ENV + cartridge (IST Ltd., Mid Glamorgan, United Kingdom) with 1 g of highly cross-linked styrene-divinyl benzene (SDVB) polymer (40–140 ím, cod. no. 915-0100-C). The cartridges were sequentially conditioned with methanol (15 mL) and distilled water (20 mL). A sample of 50 mL of wine diluted with 50 mL of distilled water and containing 0.1 mL of internal standard (4-decanol at 132 ppm in a 50% hydroalcoholic solution) was applied percolated.

The free aroma components were eluted with 30 mL of dichloromethane; the solution was dried with Na2SO4 and concentrated to 1.5 mL on a Vigreux column. Afterward, concentrated samples were stored at −10°C and, prior to GC analysis, further concentrated to 100 μL under a gentle nitrogen stream.

#### HRGC-MS Analysis

GC-MS analyses were conducted using Agilent 6890 gas chromatograph with a mass spectrometric detector (MSD) model 5973N (Agilent Technologies Deutschland Gmbh, Waldbronn, Germany).

The capillary column was an HP-Innowax (Agilent) (30 m × 0.25 mm i.d. and 0.25 μm phase thickness), and the column oven program was 35°C (2 min), at 30°C/min to 60°C (0.5 min), at 2°C/min to 160°C (10 min), and at 3°C/min to 230°C (10 min). Injector temperature was 250°C, and the injection (2 μL) was in split mode 1:5. The carrier gas was helium at 1 mL/min.

The mass spectrometer was operated in electron ionisation mode at a voltage of 70 eV. The temperatures of the source, quadrupole, and transfer line were 230, 150, and 220°C, respectively. The acquisition mass range was 30–300 amu.

The components of the wine aroma were identified comparing fragmentation patterns in the mass spectra with those stored on databases (McLafferty and Stauffer, 1991; Adams et al., 2001). Quantification was carried out by the internal standard method, so the concentration was expressed in μg/L equivalent of 4-decanol.

### Statistical Analysis

A one-way analysis of variance (ANOVA) and a Student’s *t*-test (*P* < 0.05) was carried out to check for significant differences between the biotechnological parameters of both enzymes. Meanwhile, Duncan’s test was applied to study the significant differences among the concentrations of volatile compounds released in wine when both enzymes at different concentrations were used. All statistical analyses were carried out using IBM SPSS Statistics version 24 (SPSS Inc., Chicago, IL, United States).

## Results

### Biotechnological Characterisation of Free and Immobilised β-Glucosidase

#### pH Stability

**FIGURE 1:**
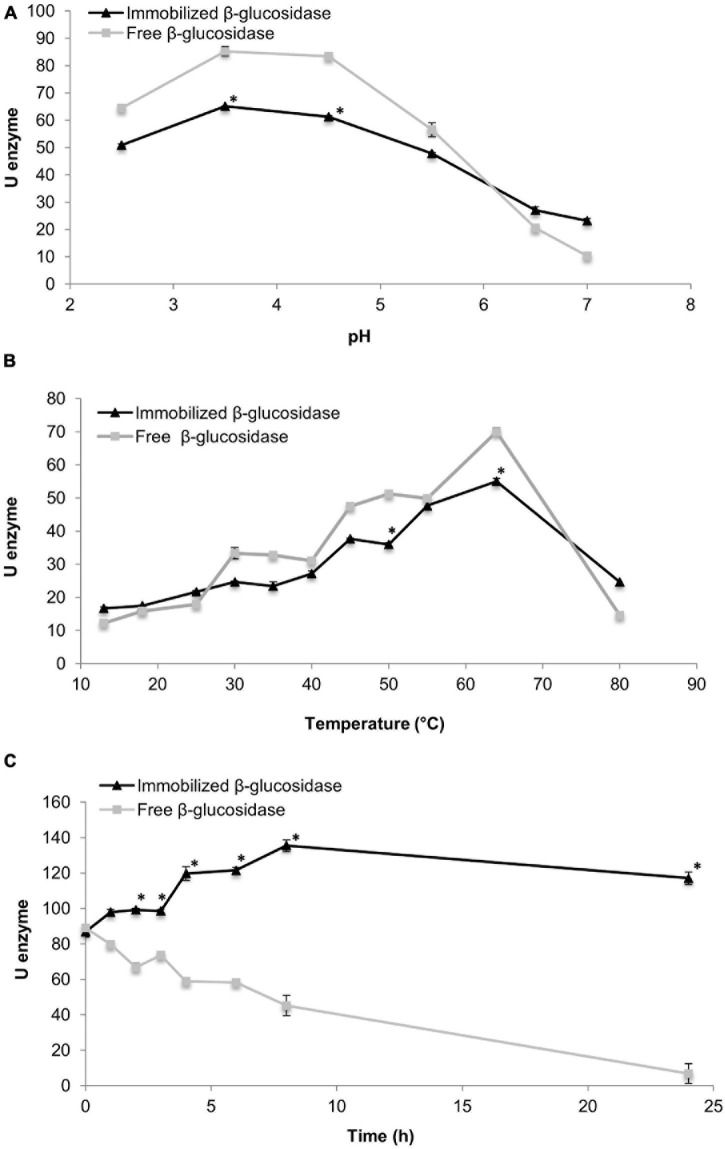
Effect of pH **(A)** and temperature **(B)** values on the activity of free and immobilised β-glucosidase and their hydrolytic capability for 24 h **(C)**. * indicate significant differences between values.

Free and immobilised enzymes were tested under diverse pH conditions [from 2.5 to 7.0 (Figure 1A)]. The two enzymes were stable between pH 2.5 and 4.5, but a slight decrement was observed at pH 5. Student’s *t*-test indicated that significant differences between enzyme units of free and immobilised β-glucosidases were observed only at pH 4 and 5, which also corresponds the optimum pH range (pH 3.5 and 4.5 for both enzymes).

#### Optimum Temperature of Hydrolysis

Temperatures ranging from 13 to 80°C were studied (Figure 1B). At the lowest temperatures (13–25°C), the immobilised β-glucosidase presented better hydrolysis capability although no significant differences were found between both enzymes at those temperatures. An increment in both activities was detected at a temperature range of 4064°C, and the maximum significant activity was reached at 64°C for both enzymes.

#### Hydrolytic Capability Along the Time

The hydrolytic activity of both enzymes was monitored for 24 h. Results for free and immobilised β-glucosidase are shown in Figure 1C. The hydrolytic activity of the immobilised enzyme increased from 0 to 8 h, and a slight decrease was observed from 8 to 24 h. Significant differences were detected in all evaluated times except at 0 and 2 h. Moreover, the highest activity value was determined at 8 h. In contrast, a reduction in the activity of the free enzyme was observed during the 24 h, especially between 8 and 24 h when the lowest activity values were observed. After 24 h, the activity of immobilised enzyme was higher than that of free form.

#### Kinetic Constants

The Km and Vmax values of the free and immobilised β-glucosidases are observed in Table 1. Similar Km values were obtained (0.22 and 0.21, respectively), which could indicate that affinity to substrate would be similar for both enzymes at these conditions (optimum pH and temperature). Nevertheless, the Vmax value was much higher for immobilised β-glucosidase (3.35 μmol min^–1^ mg^–1^).

**TABLE 1:** Apparent kinetic constants of native and immobilised β-glucosidase.

	***V*_max_^a^**	***K*_m_^b^**
Free β-glucosidase	2.52	0.22
Immobilised β-glucosidase	3.35	0.21

*^*a*^In μmol min^–1^mg^–1^.*

*^*b*^In mM.*

#### Reusability of Immobilised Enzyme

**FIGURE 2:**
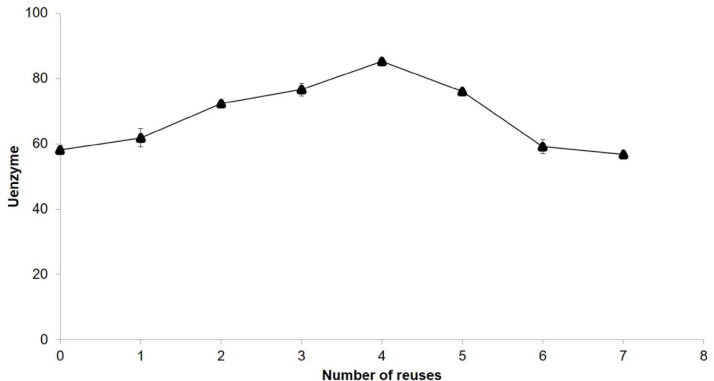
Reusability of immobilised β-glucosidase.

After reutilisation of immobilised β-glucosidase for seven times (Figure 2), the observed activity was 96.5%. Nevertheless, this enzyme showed higher values of activity during the reuse, and a peak of hydrolytic activity was reported at the fourth use.

### Enzymatic Hydrolysis of Aroma Precursors in Grape Must

**FIGURE 3:**
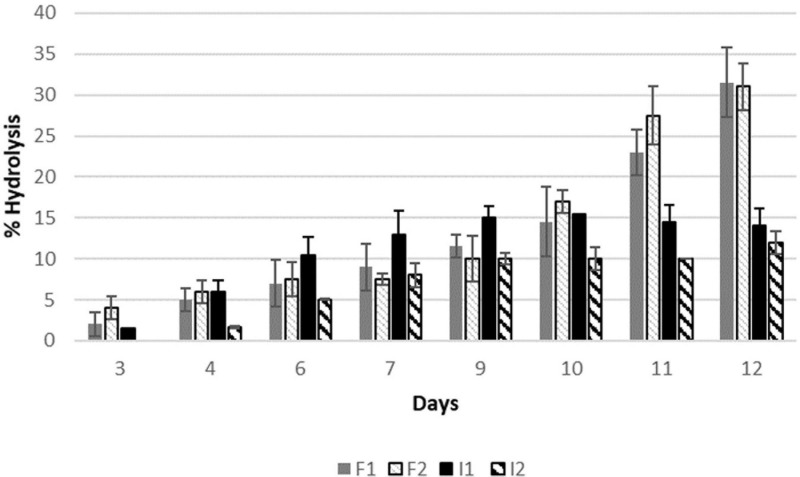
Percentage of aroma precursors hydrolysed by the enzymatic treatment of a glycosidic extract from Muscat must. F1 free enzyme 0.013 g/mL, F2 0.026 g/mL, I1 immobilised enzyme 0.013 g/mL, and I2 immobilised enzyme 0.026 g/mL.

The aroma precursors isolated from grape must were put in contact with the enzymes for 12 days at 37°C. The results of the hydrolysis of bonded compounds by free and immobilised β-glucosidase in the different doses are shown in Figure 3, where 100% corresponds to the total hydrolysis of precursors (acid hydrolysis).

It is shown that the free enzyme hydrolysing capability was the same (30%) after 12 days of treatment independently of the dose used (recommended and the half). Regarding the immobilised enzymes, hydrolysis ratio was lower, with a maximum value of 15.5% for the recommended dose. However, no significant differences were observed during the liberation of the precursors or the maximum value reached between both doses.

### Application of Immobilised and Free β-Glucosidase in White Wine

#### Aroma Precursors Hydrolysed by Free and Immobilised β-Glucosidase

The volatile compounds in wines after treatment with free and immobilised enzymes are observed in Table 2.

**TABLE 2:** Concentration of volatile compounds released by free and immobilised β-glucosidases at different doses.

	**Immobilised enzyme**		**Free enzyme**	
**Volatile compound**	**(0.013 g/mL)**	**(0.026 g/mL)**	**(0.013 g/mL)**	**(0.026 g/mL)**
Oxido a	22.61 ± 2.51^a^	46.52 ± 6.01^b^	75.54 ± 5.07^c^	108.14 ± 3.01^d^
Oxido b	21.44 ± 2.50^a^	42.40 ± 2.51^b^	71.65 ± 1.52^c^	104.90 ± 4.56^d^
Linalool	105.01 ± 5.00^a^	97.00 ± 6.56^a^	127.81 ± 4.01^b^	103.87 ± 4.00^a^
OH trienal	31.61 ± 2.05^a^	80.96 ± 3.01^c^	52.32 ± 2.51^b^	56.49 ± 3.12^b^
Alfa terpineol	59.34 ± 2.51^a^	71.88 ± 1.63^b^	77.41 ± 1.51^c^	72.58 ± 1.51^b^
Oxido c	153.29 ± 2.06^a^	256.13 ± 2.01^b^	319.16 ± 3.01^c^	392.16 ± 3.01^d^
Oxido d	53.20 ± 2.03^a^	68.04 ± 2.00^b^	85.12 ± 2.01^c^	125.13 ± 1.87^d^
Citronelol	17.66 ± 1.52^a^	25.27 ± 2.05^b^	34.39 ± 1.51^c^	35.45 ± 2.50^c^
2,7 dimetil 4,5 octanediol	9.31 ± 0.62^a^	8.16 ± 0.48^a^	13.53 ± 1.76^b^	15.42 ± 2.13^b^
Nerol	45.04 ± 2.00^c^	36.89 ± 2.59^b^	24.54 ± 2.50^a^	24.67 ± 2.51^a^
Geraniol	13.65 ± 1.52^b^	9.70 ± 0.26^a^	9.27 ± 0.75^a^	8.82 ± 0.16^a^
OH diol1	875.29 ± 8.93^b^	766.05 ± 6.12^a^	757.53 ± 3.11^a^	936.00 ± 3.60^c^
Endiol	18.18 ± 1.65^a^	20.07 ± 2.61^a^	25.39 ± 1.51^b^	30.18 ± 2.02^c^
OH diol2	59.87 ± 1.63^a^	68.06 ± 2.00^b^	78.41 ± 1.51^c^	87.74 ± 2.05^d^

*^*a*–*d*^Different superscripts in the same row indicate significant statistical differences (*P* < 0.05) according to the Duncan test from ANOVA.*

When comparing their concentrations, significant differences were observed. In no case was there a relationship between the dose of enzyme used and the concentration of compound released.

The most intergroup differences were for oxide a, oxide b, oxide c, oxide d, and oh diol2, where each sample represents a subset (*F* > 134).

The same trend was found for eight compounds: endiol, oxide a, oxide b, alfa terpineol, oxide c, oxide d, citronelol, 2,7 dimetil 4,5 octanediol, and oh diol 2. A higher concentration was observed when the free enzymes were used compared with the immobilised ones. All cases showed significant differences (*P* < 0.05) between the free and immobilised enzymes except with alpha terpineol, where the immobilised form liberated the same quantity (0.026 g/mL) as that of the free one according to the Duncan test.

The contrary effect was shown with nerol and geraniol, which were in higher concentration when the immobilised enzymes were used, although in both cases, the enzyme in a lower concentration (0.013 g/mL) achieved better results. Only OH trienal appeared in a higher concentration when the immobilised enzyme (0.026 g/mL) was used.

In the case of linalool and oh diol, a clear trend was not observed since in the first case, the best results were detected with the use of the immobilised enzyme (0.013 g/mL), and no significant differences were found between the rest of the enzymes. In the case of the second compound, the best result was obtained using the free (0.026 g/mL) and immobilised enzymes (0.013 g/mL).

## Discussion

The principal aim of this study was to evaluate the effect on aroma improvement in white wines treated with free and immobilised commercial β-glucosidases as well as to study the optimal biotechnological conditions between both enzymes.

Regarding optimal conditions, it has been reported that the optimum pH for immobilised and free β-glucosidases was the same, and some authors have even found similar values to those in the present work (pH 4) although the pH stability range may differ owing to enzyme origin (Fan et al., 2011; Ahmed et al., 2013). Nevertheless, these types of immobilised enzymes seem to show better activity (U enzyme) than free enzymes when pH is near 7 as described in this study and by Fan et al. (2011). In the case of hydrolysis temperature, an increment was observed on enzymatic activity for both β-glucosidases at around 60°C. Other authors found similar results although it was maintained until 90°C (Romo-Sánchez et al., 2014). In contrast, β-glucosidase activity, above the optimum temperature, could be gradually lost as well (Das et al., 2015). Optimum temperature and stability temperature may vary from one study to another owing to the microbial strain used for enzyme production (Busto et al., 1997).

Enzyme immobilisation has proven to be an efficient tool that could retain part of the hydrolytic capability of an enzyme even for long storage periods (Hu et al., 2018), so it is logical that hydrolytic activity loss is more accentuated in the free β-glucosidase than in the immobilised one. Furthermore, kinetic-constant results reported by Romo-Sánchez et al. (2014) were similar to those documented in the present study whereas Hernández-Maya and Cañizares-Macías (2018) and Dal Magro et al. (2019) showed that free β-glucosidase was more efficient than immobilised β-glucosidase. Catalytic efficiency may be affected after immobilisation, which could hinder the accessibility of the substrate to active sites, so the affinity of the immobilised enzyme could decrease compared with that of the free enzyme depending on the matrix and particle size used in the immobilisation procedure (Hernández-Maya and Cañizares-Macías, 2018; Dal Magro et al., 2019).

For industrial application, the immobilisation of enzymes entails significant cost benefits because it facilitates efficient recovery and reuses enzymes (Ahmed et al., 2013). The retained hydrolysis activity of some immobilised β-glucosidases after 8 uses was 51.04% (Ahmed et al., 2013), much lower compared with the results obtained in the present study. However, it could be explained by the fact that the carrier matrix used was different. Long-store reusability process (30 times) has shown that these enzymes can retain up to 30% (Hu et al., 2018) or 21% (Romo-Sánchez et al., 2014) of their original activity although it has been observed that a great percentage of the initial activity could be maintained depending on the substrate used for catalysis (Dal Magro et al., 2019). Additionally, the hydrolysis capability during time of the current commercial free β-glucosidases are subjected to temperature changes and not specified by the trading houses, so the utilisation of immobilised enzymes would standardised the whole process.

Regarding the hydrolysis of isolated aroma precursors in grape must, the same trend is observed. There is loss of activity in the immobilised forms, but it can be compensated by the advantages presented by the immobilised process. Similar results were found in related studies (Romo-Sánchez et al., 2014).

Enzymatic hydrolysis is required to enrich wine flavour by releasing free aromatic compounds from natural glycoside precursors. Concerning direct volatile compound releases on the wine, it was observed that the enzymatic treatments generally caused an expected increase in compounds from glycosidic precursors. Consequently, the use of β-glucosidase has gained momentum owing to the ability of this enzyme to catalyse transglycosylation reactions (Romo-Sánchez et al., 2014), that are of interest to the wine industry since aromatic profile of a wine is one of the essential parameters for quality determination.

The family of terpenic compounds is the basic aromatic constituent of varieties such as Muscat, among others. These compounds add floral, sweet, and citric aromas (González-Barreiro et al., 2015). These results were also obtained by other authors such as Romo-Sánchez et al. (2014) and de Ovalle et al. (2018). Particularly remarkable was the release of large amounts of nerol and, to a lesser extent, of geraniol, the amounts of which were larger when it came to immobilised enzymes as well as OH diol 1.

The enzymes, both immobilised and free, release compounds related to aroma that are beneficial to wine. Similar studies have always obtained better results when the immobilised enzyme was used (Gueguen et al., 1996; Romo-Sánchez et al., 2014; de Ovalle et al., 2018). Moreover, some authors have indicated the treatment of red wines with β-glucosidase from native yeasts could make possible to avoid the long periods of aging and allow the production of wines with sensory notes of evolution (de Ovalle et al., 2021). Immobilised forms can be of greater benefit if all of their characteristics are considered, not only the release of these compounds. They are more robust and show greater stability and resistance to environmental changes, especially against long storage periods (Romo-Sánchez et al., 2014). On the other hand, the immobilisation offers enzymes that can be reused in various processes, which entails significant cost savings in any biotechnological process, as well as the preparation of enzyme-free products, since they can easily be separated from the wine. These advantages are particularly relevant for oenological processes (González-Pombo et al., 2011).

In the past years, some questions have come up in relation to the application of this technology in the oenological industry since there are few successful examples, so is still needed of assays in different industrial and sensorial scenarios (DiCosimo et al., 2013; Espejo, 2021). Nevertheless, various authors have improved the aroma of wine during its production using immobilised enzymes (β-glucosidase, α-arabinosidase or α-rhamnosidase) and, together with this study, both support the technological advantages which brings the immobilisation of enzymes to this field (DiCosimo et al., 2013).

## Data Availability Statement

The raw data supporting the conclusion of this article will be made available by the authors, without undue reservation.

## Author Contributions

AB and MA-V designed the study. PF-P and BG-B performed the experiments and analysis. PF-P, BG-B, MA-V, and AB collected and studied the data. All authors wrote and reviewed the manuscript.

## Conflict of Interest

The authors declare that the research was conducted in the absence of any commercial or financial relationships that could be construed as a potential conflict of interest.

## Publisher’s Note

All claims expressed in this article are solely those of the authors and do not necessarily represent those of their affiliated organizations, or those of the publisher, the editors and the reviewers. Any product that may be evaluated in this article, or claim that may be made by its manufacturer, is not guaranteed or endorsed by the publisher.
